# Educational Interventions to Improve Knowledge and Attitudes Toward Human Papillomavirus (HPV) Vaccination and Cervical Cancer Screening Among Japanese University Students

**DOI:** 10.7759/cureus.75558

**Published:** 2024-12-11

**Authors:** Takeshi Fukuda, Mayuko Ueda, Rei Aida, Keiko Ota, Hisako Yoshida, Ayumi Shintani, Megumi Okada, Yukiko Takaki, Kokoro Amano, Toshiyuki Sumi

**Affiliations:** 1 Department of Obstetrics and Gynecology, Osaka Metropolitan University Graduate School of Medicine, Osaka, JPN; 2 Department of Medical Statistics, Osaka Metropolitan University Graduate School of Medicine, Osaka, JPN; 3 Center for Clinical Research and Innovation, Osaka Metropolitan University Hospital, Osaka, JPN; 4 Department of Health Promotion, Osaka City Public Health Bureau, Osaka, JPN

**Keywords:** cervical cancer, educational intervention, health belief model, human papillomavirus, vaccination

## Abstract

Aim

This study evaluates university students' knowledge and attitudes toward Human Papillomavirus (HPV) vaccination and cervical cancer screening and assesses the impact of educational interventions.

Methods

Participants from Osaka Metropolitan University, Osaka City University, and Osaka Prefecture University completed questionnaires before and after receiving educational materials, including cartoons and a video featuring medical professionals. We compared the correct answer rates for knowledge-related questions and evaluated changes in behavioral characteristics and attitudes toward HPV vaccination and cervical cancer screening before and after distributing the educational materials. The Health Belief Model (HBM) was used to measure changes in perceived susceptibility, severity, benefits, and barriers.

Results

A total of 15,061 students were invited to participate, with 234 completing the study. Significant improvements in knowledge related to HPV, cervical cancer, and associated preventative measures were observed post-intervention. The percentage of correct answers to knowledge-based questions increased across all items (P<0.01). Furthermore, positive attitudes toward HPV vaccination significantly rose, with male participants demonstrating the most substantial change (P<0.01). In terms of the HBM, perceived susceptibility and perceived benefits increased significantly among male participants (P=0.0055 and P<0.001, respectively), while perceived barriers were reduced (P<0.001). Among female participants, only the perceived benefit increased significantly (P<0.001). Most participants rated the educational materials as clear and easy to understand, reinforcing the utility of engaging and accessible content in promoting health awareness.

Conclusion

Educational interventions can effectively improve knowledge and attitudes toward HPV vaccination, potentially increasing preventative health behaviors and reducing the incidence of cervical cancer.

## Introduction

Cervical cancer ranks as the fifth most common type of cancer in terms of incidence and the fourth in mortality rates, with 604,127 new cases and 341,831 deaths reported globally in 2020 [1]. The majority of cervical cancer cases are attributed to infections by different types of high-risk human papillomaviruses (HPVs). Specifically, HPV type 16 is responsible for approximately 60% of cases, and HPV type 18 accounts for about 10% of all cervical cancer cases, followed by types 45, 31, and 33 [2]. HPV vaccination is the most effective strategy for preventing cervical cancer. Complete HPV vaccine coverage can reduce the risk of developing cervical cancer by up to 90% globally [2].

In Japan, the government started funding support for HPV vaccination in 2010, targeting girls aged 12-16, and achieved a vaccination rate of 70-80% among the intended recipients by 2012 [3]. Subsequently, in April 2013, the government incorporated HPV vaccination into the national immunization program, providing it as a publicly funded vaccine. However, merely two months later, the government halted the proactive recommendation of HPV vaccination due to multiple reports of scientifically unverified adverse events circulated by the mass media in June 2013, leading to a drastic decline in the vaccination rate to 0.6% [4]. Following surveys indicating no scientific evidence of a causal relationship between vaccination and various adverse events [5-8], the government resumed the proactive recommendation of HPV vaccination in April 2022. This included catch-up vaccination for women born between 1997 and 2005 who missed the opportunity for vaccination during the suspension of the vaccination program. However, despite these efforts, the vaccination completion rate remains low, significantly below the WHO’s recommended rate of 90% necessary for eradicating cervical cancer.

Another highly effective method for preventing cervical cancer is cancer screening. The National Cancer Institute reported that in 2021, 72.4% of women aged 21-65 years old in the U.S. underwent cervical cancer screening [9]. In contrast, a 2022 survey found that only 46.4% of participants had undergone cervical cancer screening within the past two years, a figure significantly lower than expected [10].

Given the low rates of both HPV vaccination and cervical cancer screening, it is imperative to increase these rates in Japan to reduce the incidence of cervical cancer. Encouraging higher participation in both HPV vaccination programs and regular cervical cancer screening can significantly positively impact the national effort to diminish the prevalence and mortality associated with this disease.

This study aims to assess the current knowledge and attitudes regarding HPV vaccination and cervical cancer screening among university students through the utilization of a questionnaire. Additionally, it seeks to evaluate the impact of educational interventions, including cartoons that provide information about HPV vaccination and cervical cancer and a video featuring medical professionals discussing cervical cancer and emphasizing the significance of cervical cancer screening in improving knowledge and attitudes towards these preventative measures.

## Materials and methods

Participants for this study were recruited from students attending Osaka Metropolitan University, Osaka City University, and Osaka Prefecture University. Invitations to participate were sent via email to all students, irrespective of sex, using email addresses provided by the respective universities. Participants were assured that their involvement was voluntary and would not impact their academic standing. Informed consent for participation was obtained electronically through a link included in the invitation email. Participants retained the right to withdraw from the study at any time, even after initially agreeing to participate. Their affiliations with the universities could be determined through their email addresses.

We employed a questionnaire designed to assess various factors, including demographics (such as age, sex, state of residence, and marital status), knowledge, and behavioral tendencies related to cervical cancer, cervical cancer screening, HPV, and HPV vaccination, as well as attitudes toward cervical cancer screening and HPV vaccination. To accommodate sex differences in knowledge and attitudes, we developed two versions of the questionnaire: one for female students and another for male students. Appendix 1 illustrates the questions posed in both questionnaires.

Students were asked to fill out the questionnaire on two occasions: once before and once after they were provided with the educational materials. The distribution and collection of responses for the questionnaire were conducted entirely online using the REDCap (Vanderbilt University, Nashville, Tennessee) platform [11, 12].

We assessed the correlation between participants’ characteristics and the accuracy of their responses to knowledge-related questions before the distribution of the educational materials. We also conducted comparisons of the correct answer rates for knowledge-related questions and evaluated changes in behavioral characteristics and attitudes toward HPV vaccination and cervical cancer screening before and after the distribution of the educational materials. Additionally, the clarity and comprehensibility of the educational materials were gauged through a specific question included at the end of the questionnaire, following the distribution of the materials.

The Health Belief Model (HBM) was employed to evaluate behavioral characteristics toward HPV vaccination and cervical cancer screening. The HBM comprises six constructs: perceived susceptibility, perceived severity, perceived benefits, perceived barriers, cues to action, and self-efficacy [13]. Our study focused on assessing four key HBM constructs: perceived susceptibility, perceived severity, perceived benefits, and perceived barriers. Table 1 delineates which questionnaire items correspond to these HBM constructs.

**Table 1:** Correspondence table of HBM components and questions. HBM, health belief model; HPV, human papillomavirus; OB/GYN, obstetrician and gynecologist.

HBM Component	Question
Perceived susceptibility	Do you think you could be at risk of developing it/ them? (male and female)
Perceived severity	Do you think that some of them are life-threatening disease? (male)
	Do you think that it is a life-threatening disease? (female)
	Do you think that developing cervical cancer means you cannot conceive? (female)
Perceived benefit	Do you think that they are preventable with the HPV vaccine? (male)
	Do you think that the HPV vaccine is effective in preventing cervical cancer? (female)
	Do you think that cervical cancer screening can prevent or detect cervical cancer in its early stages? (female)
Perceived barriers	Would you receive the HPV vaccine if it were free or cheaper? (male)
	Do you think that you have concerns about getting the HPV vaccine? (female)
	Do you think that you feel hesitant or resistant to visiting an OB/GYN for cervical cancer screening? (female)
	Do you think that you are unwilling to undergo cervical cancer screening because you do not know what kind of tests will be performed? (female)
	Do you think that undergoing regular cervical cancer screening is a hassle? (female)

Answers were scored on a scale from 1-5, ranging from 'I strongly agree' to 'I strongly disagree.' Changes in HBM constructs were gauged using the median score for each construct, comparing these median scores before and after the educational materials were provided. For constructs assessed by multiple questions, an average score of all questions for each participant was calculated for comparison. Furthermore, we examined the relationship between the degree of change in each HBM construct score and the shifts in attitudes toward HPV vaccination for both males and females, as well as cervical cancer screening for females, before and after the educational materials were provided.

The educational materials were distributed to participants via email in four weekly installments, each comprising different content aimed at improving understanding and awareness of HPV, its vaccination, and cervical cancer screening. The first educational piece was a cartoon explaining the mechanism of HPV infection in humans (Appendices 2-3). This cartoon aims to provide basic knowledge about the nature of HPV and how it affects the human body.

The second educational piece was a cartoon highlighting the significance of HPV vaccination for males (Appendices 4-5), accompanied by a web link to the Ministry of Health, Labour and Welfare’s announcement regarding catch-up vaccination (https://www.mhlw.go.jp/stf/seisakunitsuite/bunya/kenkou/hpv_catch-up-vaccination.html, accessed November 11, 2024). This material focuses on encouraging HPV vaccination among males, addressing its benefits and the available vaccination programs.

The third educational piece was a cartoon about cervical cancer screening, HPV, and cervical cancer (Appendices 6-10), along with a web link to a quiz on cervical cancer and HPV (https://minpapi.jp/quiz/, accessed November 11, 2024). This interactive component is designed to engage participants in learning more about cervical cancer and HPV in an engaging and informative way.

The fourth educational piece was a video featuring a medical professional (who is also one of the authors of this manuscript) discussing cervical cancer and the importance of cervical cancer screening, available publicly on YouTube (https://www.youtube.com/watch?v=awWY4F1Btbg, accessed November 11, 2024). This video aims to provide credible and professional insight into the importance of regular cervical cancer screenings.

All cartoons were produced by the Minpapi Association and were used with permission for this study. The sequence and content of these materials were strategically designed to progressively educate participants on HPV and cervical cancer, encouraging informed decisions regarding HPV vaccination and cervical cancer screening.

For descriptive statistics, categorical variables are presented as frequencies and proportions; continuous variables are presented as means and inter-quartile ranges. The correct answer rates of the baseline survey were aggregated according to the participants’ characteristics. McNemar’s tests were used to compare knowledge-related questions and attitudes toward HPV vaccination and cancer screening before and after the distribution of the educational materials. We analyzed the influence of changes in each sub-score of the HBM on behavioral changes, such as HPV vaccination and cervical cancer screening, after learning through the educational materials using logistic models. A two-sided P-value of P<0.05 was considered to indicate a statistically significant difference. All statistical analyses were performed using R version 4.2.2 (R Foundation for Statistical Computing, Vienna, Austria).

## Results

**Figure 1:**
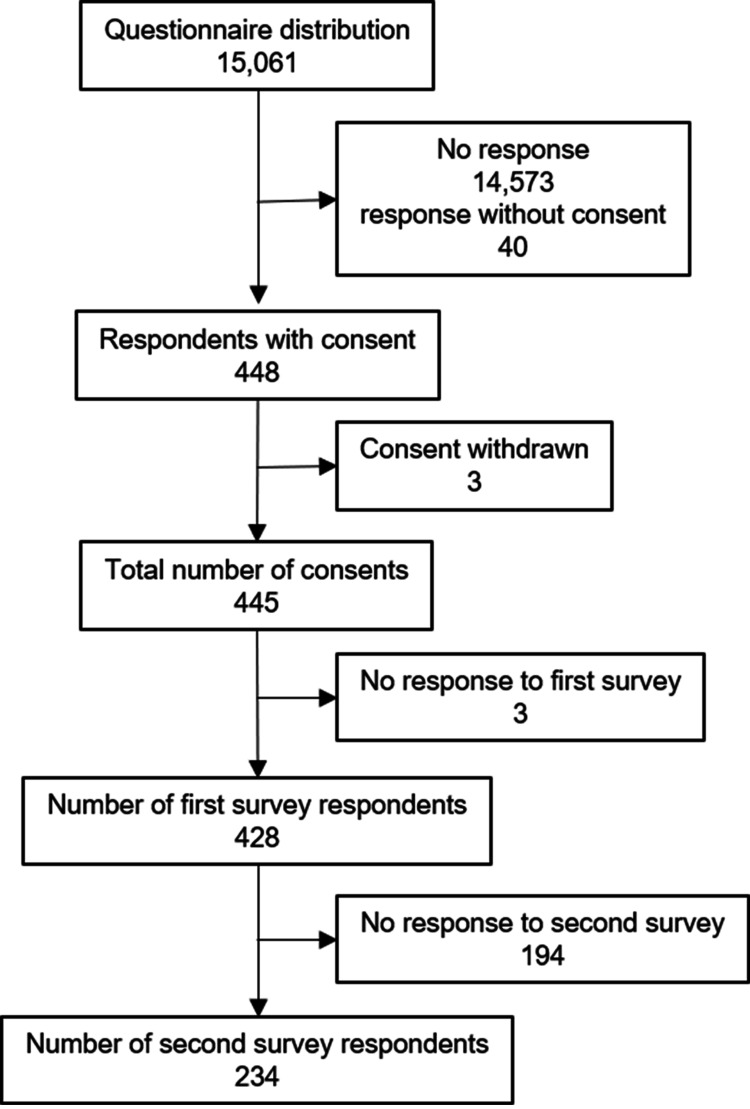
Response status following the distribution of the questionnaires.

The questionnaires, distributed to a total of 15,061 students, yielded 234 complete responses. The response status following the distribution is shown in Figure 1.

Participant demographics are detailed in Table 2. The participant pool consisted of 69 males and 165 females, with a median age of 21 years, spanning from 18 to 53 years old. When it comes to living arrangements, 29.1% of participants reported being single, 65.0% lived with their parents, and 6.0% lived with individuals other than their parents. Regarding relationship status, a significant majority, 73.9%, were single without a partner, 23.5% were not married but had a partner, and a small fraction, 2.6%, were married. The academic backgrounds of participants varied, with 10.3% studying medicine, 17.9% in nursing, and the remaining 71.8% pursuing other fields. The breakdown of participants by sex is detailed in the table.

**Table 2:** Participant characteristics. ^a^Median

Characteristics	Male, n=69	Female, n=165	All, n=234
Age, years^a^	21 (18-48)	21 (18-53)	21 (18-53)
Residency status, n (%)			
Living alone	18 (26.1%)	50 (30.3%)	68 (29.1%)
Living with parents	49 (71.0%)	103 (62.4%)	152 (65.0%)
Living with others (not parents), n (%)	2 (2.9%)	12 (7.3%)	14 (6.0%)
Marital status, n (%)			
Single (not in a relationship)	53 (76.8%)	120 (72.7%)	173 (73.9%)
Single (in a relationship)	15 (21.7%)	40 (24.2%)	55 (23.5%)
Married	1 (1.4%)	5 (3.0%)	6 (2.6%)
Major, n (%)			
Medicine	7 (10.1%)	17 (10.3%)	24 (10.3%)
Nursing	2 (2.9%)	40 (24.2%)	42 (17.9%)
Others	60 (87.0%)	108 (65.5%)	168 (71.8%)

Table 3 illustrates the correlation between the characteristics of the participants and the accuracy of their responses before the distribution of educational materials.

**Table 3:** Accuracy rate of knowledge-related questions prior to distribution of educational materials by participant characteristics.

	Characteristics	n	n (%) of correct responses
Please select the items you think are related to the development of cancer.			
	Age, years		
	Less than 25 years old	201	115 (57.2%)
	Over 25 years old	33	29 (87.9%)
	Residency status		
	Living alone	68	47 (69.1%)
	Living with parents	152	84 (55.3%)
	Living with others (not parents)	14	13 (92.9%)
	Marital status		
	Single (not in a relationship)	173	97 (56.1%)
	Single (in a relationship)	55	42 (76.4%)
	Married	6	5 (83.3%)
	Major		
	Medicine	24	19 (79.2%)
	Nursing	42	35 (83.3%)
	Others	168	90 (53.6%)
Which sexes can be affected by the human papillomavirus (HPV) or develop related diseases?			
	Age, years		
	Less than 25 years old	201	108 (53.7%)
	Over 25 years old	33	23 (69.7%)
	Residency status		
	Living alone	68	41 (60.3%)
	Living with parents	152	81 (53.3%)
	Living with others (not parents)	14	9 (64.3%)
	Marital status		
	Single (not in a relationship)	173	91 (52.6%)
	Single (in a relationship)	55	36 (65.5%)
	Married	6	4 (66.7%)
	Major		
	Medicine	24	15 (62.5%)
	Nursing	42	29 (69.0%)
	Others	168	87 (51.8%)
What is the primary mode of transmission for human papillomavirus (HPV)?			
	Age, years		
	Less than 25 years old	201	143 (71.1%)
	Over 25 years old	33	28 (84.8%)
	Residency status		
	Living alone	68	49 (72.1%)
	Living with parents	152	111 (73.0%)
	Living with others (not parents)	14	11 (78.6%)
	Marital status		
	Single (not in a relationship)	173	125 (72.3%)
	Single (in a relationship)	55	41 (74.5%)
	Married	6	5 (83.3%)
	Major		
	Medicine	24	21 (87.5%)
	Nursing	42	39 (92.9%)
	Others	168	111 (66.1%)
Who is eligible to receive a publicly funded (free) HPV vaccine for human papillomavirus (HPV) infection?			
	Age, years		
	Less than 25 years old	201	114 (56.7%)
	Over 25 years old	33	22 (66.7%)
	Residency status		
	Living alone	68	42 (61.8%)
	Living with parents	152	83 (54.6%)
	Living with others (not parents)	14	11 (78.6%)
	Marital status		
	Single (not in a relationship)	173	101 (58.4%)
	Single (in a relationship)	55	31 (56.4%)
	Married	6	4 (66.7%)
	Major		
	Medicine	24	16 (66.7%)
	Nursing	42	35 (83.3%)
	Others	168	85 (50.6%)
Are you familiar with catch-up vaccination for the HPV vaccine?			
	Age, years		
	Less than 25 years old	201	48 (23.9%)
	Over 25 years old	33	12 (36.4%)
	Residency status		
	Living alone	68	20 (29.4%)
	Living with parents	152	37 (24.3%)
	Living with others (not parents)	14	3 (21.4%)
	Marital status		
	Single (not in a relationship)	173	43 (24.9%)
	Single (in a relationship)	55	15 (27.3%)
	Married	6	2 (33.3%)
	Major		
	Medicine	24	14 (58.3%)
	Nursing	42	18 (42.9%)
	Others	168	28 (16.7%)

Except for the question about catch-up vaccination, “Are you familiar with catch-up vaccination for the HPV vaccine?”, the characteristics 'Over 25 years old,' 'Living with others (not parents),' 'Married,' and 'Majoring in medicine or nursing' were associated with a higher accuracy rate. For the question about catch-up vaccination, the characteristics 'Over 25 years old,' 'Living alone,' 'Married,' and 'Majoring in medicine or nursing,' were associated with a higher accuracy rate.

The assessment of cervical cancer and HPV vaccine knowledge was conducted using questionnaires. We examined the increase in knowledge by comparing the rates of correct answers before and after the distribution of educational materials. Regarding the question, “Please select the items you think are related to the development of cancer (multiple answers allowed),” the answer was considered correct if HPV was selected. This is because the educational materials in this study focused exclusively on HPV, and our aim was to assess knowledge specifically about HPV. Table 4 illustrates the comparison of correct answer rates to knowledge-based questions before and after the educational intervention. Significantly improved rates were observed across all questions, indicating the effectiveness of the educational materials in enhancing the participants’ understanding.

**Table 4:** Comparisons of correct answer rates for knowledge-related questions before and after the distribution of educational materials. ^a^McNemar’s test.

Question	Rate of a correct answer, n (%)		P-value
	Before	After	
Please select the items you think are related to the development of cancer.			
Male	34 (49.3%)	63 (91.3%)	<0.001^a^
Female	110 (66.7%)	135 (81.8%)	<0.001^a^
All	144 (61.5%)	198 (84.6%)	<0.001^a^
Which sexes can be affected by the human papillomavirus (HPV) or develop related diseases?			
Male	36 (52.2%)	64 (92.8%)	<0.001^a^
Female	95 (57.6%)	146 (88.5%)	<0.001^a^
All	131 (56.0%)	210 (89.7%)	<0.001^a^
What is the primary mode of transmission for human papillomavirus (HPV)?			
Male	42 (60.9%)	67 (97.1%)	<0.001^a^
Female	129 (78.2%)	162 (98.3%)	<0.001^a^
All	131 (56.0%)	210 (89.7%)	<0.001^a^
Who is eligible to receive a publicly funded (free) HPV vaccine for human papillomavirus (HPV) infection?			
Male	24 (34.8%)	56 (81.2%)	<0.001^a^
Female	112 (67.9%)	136 (82.4%)	<0.001^a^
All	136 (58.1%)	192 (82.1%)	<0.001^a^
Are you familiar with catch-up vaccination for the HPV vaccine?			
Male	5 (7.2%)	30 (43.5%)	<0.001^a^
Female	55 (33.3%)	97 (58.8%)	<0.001^a^
All	60 (25.6%)	127 (54.3%)	<0.001^a^

The assessment of attitudes toward HPV vaccination relied on participants’ responses to the question: “Have you been vaccinated against HPV?”. Responses indicating 'Completed multiple vaccinations,' 'Have been vaccinated but have not yet completed,' and 'Have never been vaccinated but would like to be vaccinated in the future' were categorized as reflecting a positive attitude. Conversely, responses of 'Have never been vaccinated and do not want to be vaccinated in the future' and 'Not sure' were classified as indicative of a negative attitude. Table 5 presents the changes in the positive attitude toward HPV vaccination before and after the distribution of educational materials. It was noted that the rate of positive attitudes toward HPV vaccination increased significantly for males, females, and all participants, indicating a favorable impact of the educational intervention.

**Table 5:** Changes in positive attitudes toward HPV vaccination before and after the distribution of educational materials. ^a^McNemar’s test HPV: Human Papillomavirus.

Sex	Before, n (%)	After, n (%)	P-value
Male	24 (34.8%)	52 (75.4%)	<0.001^a^
Female	108 (65.5%)	129 (78.25%)	<0.001^a^
All	132 (56.45%)	181 (77.45%)	<0.001^a^

The assessment of attitudes toward cervical cancer screening among females was based on responses to the question: “Have you ever been screened for cervical cancer?”. Responses indicating 'I regularly undergo it,' 'I have undergone it,' and 'I have not undergone it but would like to undergo one in the future' were considered indicative of a positive attitude. Conversely, responses of 'I have never undergone it and do not want to undergo one in the future' and 'Not sure' were categorized as reflecting a negative attitude. The rate of positive attitudes toward cervical cancer screening decreased from 143 (86.7%) before the intervention to 133 (80.6%) after the intervention, with a statistically significant P-value of 0.0129 as determined by McNemar’s test.

The behavioral characteristics were evaluated using the HBM. The median scores of each HBM component were compared before and after the distribution of the educational materials to assess changes in behavioral characteristics toward HPV vaccination and cervical cancer screening. Table 6 illustrates the change in the median score of each HBM component before and after the distribution of the educational materials. Regarding perceived susceptibility, the median score increased significantly among males. However, there was no statistically significant difference in the median score for perceived severity among both males and females before and after the distribution of educational materials. Conversely, the median score for perceived benefits increased significantly among both males and females. Finally, concerning perceived barriers, the median score increased significantly among males. These findings suggest a positive impact of the educational intervention on perceived susceptibility and perceived benefits among males, as well as on perceived benefits among females.

**Table 6:** Change in the median scores of each HBM component before and after the distribution of educational materials. HBM: Health belief model; IQR: Interquartile range. ^a^Wilcoxon signed-rank test.

HBM component	Score (median, [IQR], (range))		P-value
	Before	After	
Perceived susceptibility			
Male	4.0 [3.0, 4.0] (2.0-5.0)	4.0 [4.0, 5.0] (1.0-5.0)	0.0055^a^
Female	4.0 [4.0, 5.0] (2.0-5.0)	5.0 [4.0, 5.0] (1.0-5.0)	0.1696^a^
Perceived severity			
Male	4.0 [4.0, 5.0] (2.0-5.0)	4.0 [4.0, 5.0] (2.0-5.0)	0.1467^a^
Female	4.5 [3.8, 4.5] (2.0-5.0)	4.5 [3.5, 4.5] (1.5-5.0)	0.3720^a^
Perceived benefit			
Male	3.0 [3.0, 4.0] (1.0-5.0)	5.0 [4.0, 5.0] (1.0-5.0)	<0.001^a^
Female	4.5 [4.0, 5.0] (2.5-5.0)	5.0 [4.5, 5.0] (3.5-5.0)	<0.001^a^
Perceived barriers			
Male	4.0 [4.0, 5.0] (1.0-5.0)	5.0 [4.0, 5.0] (2.0-5.0)	<0.001^a^
Female	3.2 [2.8, 3.8] (1.5-5.0)	3.0 [2.5, 3.8] (1.0-5.0)	0.2257^a^

Table 7 presents the responses to the question assessing the understandability of the educational materials. A significant majority of participants rated the material as either 'Very easy to understand' or 'Easy to understand,' comprising 92.3% of the total responses.

**Table 7:** Evaluation of the clarity and comprehensibility of educational materials.

Response	Male, n (%)	Female, n (%)	All, n (%)
Very easy to understand	31 (44.9%)	59 (35.8%)	90 (38.5%)
Easy to understand	37 (53.6%)	89 (53.9%)	126 (53.8%)
Neither easy nor difficult	0 (0.0%)	11 (6.7%)	11 (4.7%)
A little difficult to understand	1 (1.4%)	2 (1.2%)	3 (1.3%)
I haven’t read at all	0 (0.0%)	4 (2.4%)	4 (1.7%)

## Discussion

Educational interventions have proven to be effective tools for increasing knowledge and awareness about cervical cancer and HPV [14]. Several studies have highlighted the barriers to cervical cancer screening and HPV vaccination, primarily due to limited knowledge and awareness [14-20]. The current study evaluated the effectiveness of educational interventions in enhancing knowledge and attitudes regarding HPV vaccination and cervical cancer screening among university students in Japan. The interventions, comprising cartoons and a video featuring medical professionals, aimed to improve understanding and promote preventative behaviors.

The findings indicate a significant improvement in knowledge as a result of the educational interventions. Participants demonstrated higher correct response rates for knowledge-related questions about HPV and cervical cancer following the intervention. This outcome is consistent with previous studies, such as those by Shin et al. [21] and Dönmez et al. [22], which highlighted that educational efforts effectively increase knowledge and awareness among university students, leading to healthier behaviors. Additionally, there was a notable increase in positive attitudes towards HPV vaccination among all participant groups after the educational intervention. This is particularly significant given the previously low vaccination rates in Japan due to misinformation and lack of awareness. The interventions appear to have successfully addressed some of the barriers identified in earlier research. For example, Somera et al. [20] emphasized that improved knowledge plays a crucial role in influencing vaccination behavior, a finding that is echoed in this study. In contrast, positive attitudes towards cervical cancer screening among female participants significantly decreased post-intervention. Despite this decline, the initial positive attitude rate of 86.7% prior to the distribution of educational materials was notably higher than the 64.1% reported in a previous study conducted in Japan [14]. Given the already high rate of positive attitudes among participants before the intervention, further increasing this rate poses a challenge. Moreover, even though the positive rate declined after the intervention, the positive attitude rate remained at 80.6%, which is still higher than the post-education rate of 75.2% reported in another study [14]. Therefore, the effectiveness of the educational materials used in this study in changing attitudes towards cervical cancer screening should be evaluated in populations with a lower baseline positive attitude compared to the participants in this study. The HBM analysis revealed significant increases in perceived susceptibility and perceived benefit scores, particularly among male participants. This suggests that the interventions effectively increased the perceived risk associated with cervical cancer and the perceived benefits associated with HPV vaccination. However, there was no significant change in perceived severity and perceived barriers scores, indicating areas for improvement in future educational efforts. This outcome supports the findings of Hayes et al. [23], which highlight the critical role of health beliefs in influencing vaccination behaviors. Lastly, the majority of participants rated the educational materials as clear and easy to understand, underscoring the importance of well-designed and accessible educational content. The positive reception of the materials is crucial for the effectiveness of such interventions.

The significant improvement in knowledge and positive shift in attitudes towards HPV vaccination underscore the potential of educational interventions to promote preventative health behaviors. Given the low rates of both HPV vaccination and cervical cancer screening in Japan, increasing educational outreach could significantly impact public health outcomes.

This study has several limitations. First, the response rate was relatively low, which may cause selection biases and limit the generalizability of the findings. Additionally, the reliance on self-reported data can introduce response biases. Furthermore, the target population consisted solely of university students, which does not reflect the general population. Moreover, the current study did not include follow-up assessments to measure long-term behavioral changes. However, this study uniquely integrates multimedia educational interventions, such as cartoons and videos, to improve knowledge and attitudes toward HPV vaccination and cervical cancer screening, offering an innovative and engaging method to address public health challenges. This represents a novel approach and one of the key strengths of the study. Future research should include a more generalized population and incorporate follow-up assessments to measure long-term behavioral changes, providing more robust data on the impact of these interventions.

## Conclusions

This study highlights the potential of educational interventions to improve knowledge and attitudes toward HPV vaccination among university students in Japan. Enhancing the effectiveness of such interventions is essential for increasing participation in preventative health behaviors, ultimately reducing the incidence and mortality rates of cervical cancer.

## Figures and Tables

**Figure 2:**
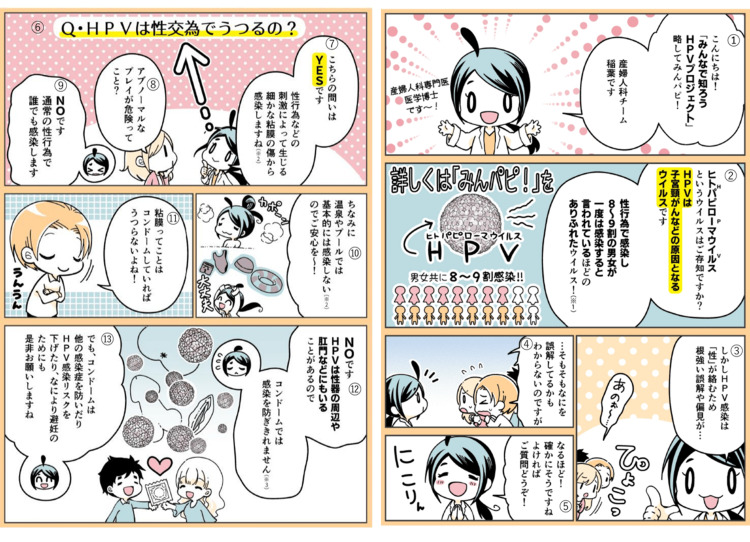
The mechanism of HPV infection in humans (1/2). 1. Hi, everyone. I'm Dr. Inaba, representing the OB/GYN department within our project team, “Minpapi”. 2. Have you heard about the human papillomavirus, commonly known as HPV? This virus is the leading cause of cervical cancer. It's transmitted through sexual contact and is so widespread that 80-90% of adults are believed to contract it at some point in their lives. 3. Yet, discussing HPV often brings about undue stigma and misconceptions due to its association with sexual health. 4. I'm not even sure what aspects I've misunderstood initially. 5. Alright. I understand. Please, don't hesitate to raise any questions you might have. 6. Q. Is HPV transmitted through sexual activities? 7. Yes, it spreads via small injuries to the mucosal lining, which can occur during sexual activities. 8. So, does engaging in unconventional sexual practices increase the risk of contracting HPV? 9. No, it is not limited to unconventional practices. HPV can infect anyone engaging in standard sexual intercourse. 10. By the way, you can rest assured that you are basically not infected by using a hot spring or swimming pool. 11. If it's transmitted through mucous membranes, it's not contagious if you use a condom, right? 12. Actually, since HPV can be found in the genital area and anus, condoms cannot prevent infection. 13. But condoms can prevent other infections and lower the risk of HPV infection. And above all, condoms should definitely be used for contraceptive purposes. Note: The numbers correspond to the translation of the similarly numbered panels.

**Figure 3:**
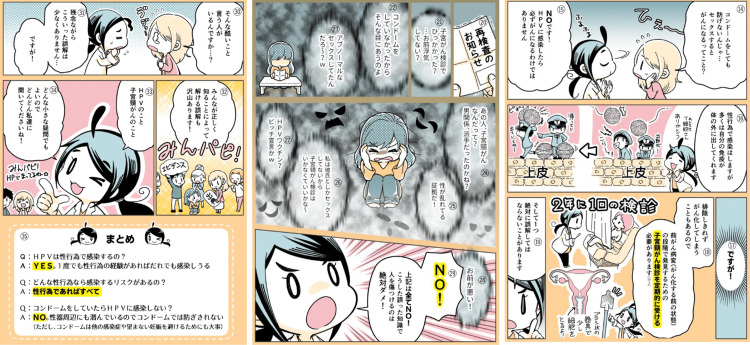
The mechanism of HPV infection in humans (2/2). 14. If using a condom does not protect against infection, does that mean that having sex can cause cancer? 15. No. HPV infection does not always result in cancer. 16. It is sexually transmitted, but is mostly eliminated by the immune system. 17. But… 18. Since it cannot always be eliminated and can cause cancer, regular cervical cancer screening is necessary to detect the disease in its precancerous stage. 19. And this is one thing that should never be misunderstood 20. Notice of Re-examination. 21. Positive for the cervical cancer? Are you having an affair? 22. This happened because you didn’t use a condom. 23. You must have had abnormal sexual activity. 24. I heard that she had cervical cancer. I wonder if she has many sexual partners. 25. That's proof of sexual promiscuity. 26. I only have sexual intercourse with my boyfriend, so I don't have to get a cervical cancer screening. 27. HPV vaccine? Is that a declaration of promiscuity? 28. It's your fault. 29. No. These are all wrong. We should never hurt people with this kind of misconceptions. 30. Who would say such a terrible thing? 31. Unfortunately, this misunderstanding is not uncommon. But! 32. There are many misunderstandings that can be cleared up when everyone is properly informed. 33. HPV and cervical cancer. 34. Ask us any questions you may have, no matter how small they may seem. 35. Summary. Q: Is HPV sexually transmitted? A: Yes. Anyone who has had sexual activity at least once can become infected. Q: What kind of sexual activity puts me at risk for infection? A: Any sexual activity. Q: Does using a condom prevent HPV infection? A: No. Since HPV can be found in the genital area and anus, condoms cannot protect against  infection. (But, condom use is also important to avoid other infections and unwanted pregnancy.) Note: The numbers correspond to the translation of the similarly numbered panels.

**Figure 4:**
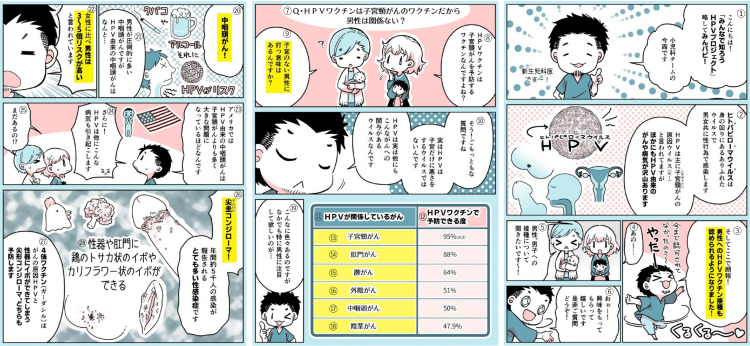
The importance of HPV vaccination for males (1/2) 1. Hi, everyone. I'm Dr. Imanishi, representing the pediatrics department within our project team, “Minpapi”. 2. HPV is a common virus that is all around us and is sexually transmitted by both men and women. HPV is said to be the virus that mainly causes cervical cancer, but there are many other types of cancer and diseases that can be caused by HPV. 3. And here's the good news. HPV vaccinations can now be given to men. 4. Well. 5. I would like to ask about HPV vaccination for men. 6. Oh, I am glad you are interested. Ask me any questions you want! 7. Are HPV vaccines relevant for men given it is a vaccine to prevent cervical cancer? 8. The HPV vaccine prevents cervical cancer, correct? 9. Is there any point in giving the HPV vaccine to men if they don’t have a uterus? 10. That's a fair question! In fact, HPV does not only affect the uterus. It is actually a virus that is associated with the following other cancers. 11. HPV-associated cancers 12. The percentage preventable by the HPV vaccine 13. Cervical cancer 14. Anal cancer 15. Vaginal cancer 16. Vulvar cancer 17. Oropharyngeal cancer 18. Penile cancer 19. There are many different types of cancer, but one in particular that men should pay attention to is 20. Oropharyngeal cancer 21. The overwhelming majority of oropharyngeal cancers occur among men, and what’s more, 22. The risk of HPV-related oropharyngeal cancers is said to be 3-5 times higher in men than in women. 23. In the United States, HPV-related oropharyngeal cancer is more common than cervical cancer and is a major problem. 24. In addition, HPV can cause other diseases. 25. Are there more diseases? 26. Condyloma acuminatum. It is a very common sexually transmitted disease, with approximately 5,000 infections reported annually. 27. The quadrivalent vaccine (Gardasil) protects against HPV infection, which causes cancer and condyloma acuminatum. 28. Cristate or cauliflower-like warts on the genitals or anus.

**Figure 5:**
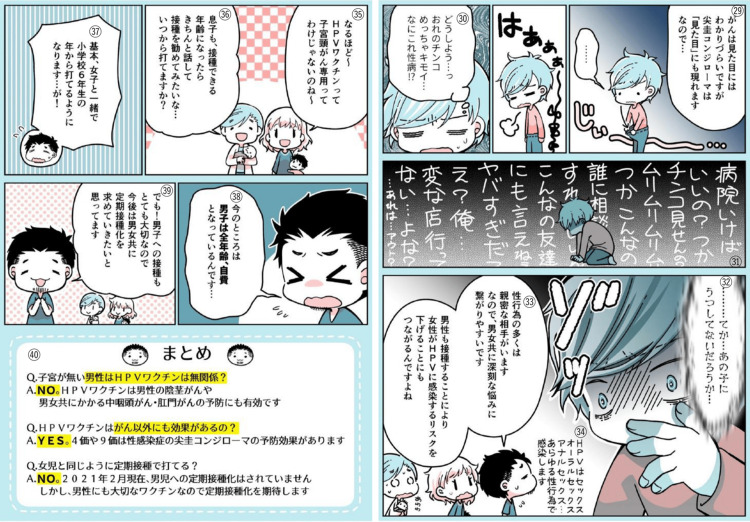
The importance of HPV vaccination for males (2/2) 29. While cancer is not visually obvious, condyloma acuminatum is visually apparent. 30. What should I do? My penis is so gross. Is this an STD? 31. Should I see a doctor? But I can't show my penis to the doctor. Who do I talk to about it? I can't even talk to my friends about it. This is a big deal, right? 32. Wait a minute. I wonder if I have transmitted it to her. 33. Most sexual activity involves an intimate partner. So, it can easily lead to serious problems for both men and women. Men can also help reduce the risk of HPV infection in women by getting the HPV vaccine. 34. HPV can be transmitted through normal sex, oral sex, anal sex, or any sexual activity. 35. I see, so the HPV vaccine is not exclusive to cervical cancer. 36. When my son is old enough to be vaccinated, I would like to discuss and recommend that he be vaccinated. When can boys be vaccinated? 37. Basically, the same as for girls, vaccination is available from the 6th grade. But! 38. At the moment, boys are vaccinated at their own expense for all ages. 39. However, since vaccination for boys is also very important, I would like to advocate for a routine immunization program for both men and women in the future. 40. Summary. Q: Is the HPV vaccine irrelevant for men since they don’t have a uterus? A: No. The HPV vaccine is also effective in preventing penile cancer, oropharyngeal cancer, and anal cancer. Q: Is the HPV vaccine effective against diseases other than cancer? A: Yes. Quadrivalent and nine-valent vaccines are also effective in preventing condyloma acuminatum. Q: Can boys be vaccinated as part of the routine immunization program as well as girls? A: No. As of February 2021, there is no routine vaccination program for boys. However, we hope that the HPV vaccine will become a part of the vaccination program since it is important for boys as well. Note: The numbers correspond to the translation of the similarly numbered panels.

**Figure 6:**
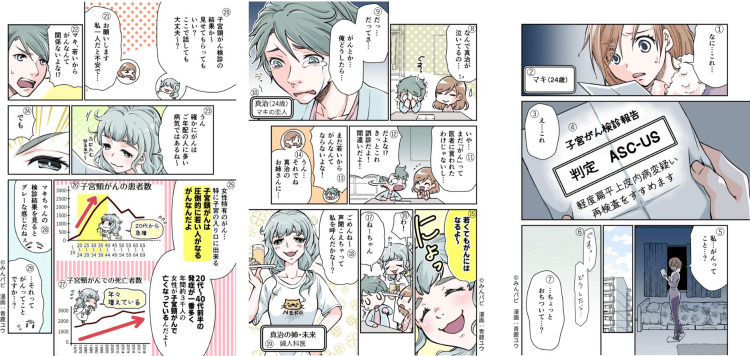
Cervical cancer screening, HPV, and cervical cancer (1/5) 1. What’s this? 2. Maki (24 years old) 3. Oh, this means… 4. The results of cervical cancer screening. ASC-US. 5. I have cancer? 6. What should I do? 7. Hey, calm down. 8. Why are you crying? 9. Because I don't know what to do now that I know I have cancer. 10. Shinji (24 years old) Maki’s boyfriend. 11. Well, I haven't been told by a doctor that I have cancer yet. 12. Right. I'm sure this is a misdiagnosis. It should be a mistake. 13. You're still young, so you don't get cancer, right? 14. So I think I'll ask your sister. 15. You can get cancer even if you're young. 16. Wow! 17. Sis! 18. I'm sorry. I heard your voice. I wonder if you called me. 19. Shinji’s sister/ Mirai (Gynecologist) 20. Cervical cancer screening results, huh? Can I see it? Can I talk to you here? 21. Please. I feel uneasy listening to you alone. 22. Maki is young, so cancer shouldn't be a concern for her, right? 23. Yeah. It is true that cancer is more common among older people. 24. But, 25. Cervical cancer, which is exclusive to women, stands out as the most prevalent cancer among younger individuals. Peak incidence occurs in women aged in their 20s to early 40s, resulting in the death of 3,000 women to cervical cancer annually. 26. Number of cervical cancer patients 27. Number of deaths from cervical cancer 28. Your cervical cancer screening results appear to be uncertain. 29. Does that mean cancer? Note: The numbers correspond to the translation of the similarly numbered panels.

**Figure 7:**
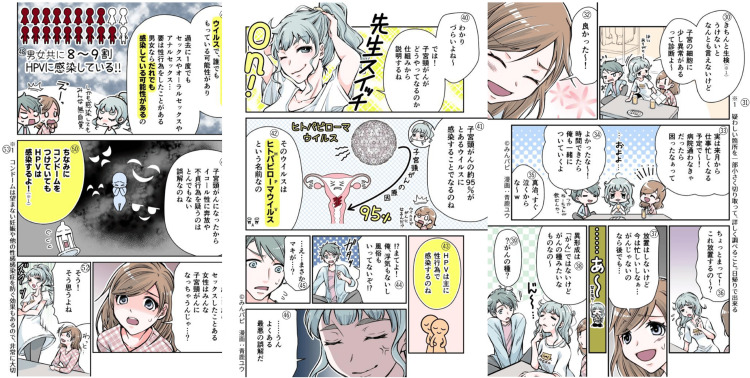
Cervical cancer screening, HPV, and cervical cancer (2/5) 30. I can't say for sure without a proper biopsy, but the diagnosis is that there is a slight abnormality in the cells of the cervix. 32. That’s good. 33. Actually, I'll be occupied with work from next month onwards, so I would be concerned if I needed to go to the hospital frequently. 34. Good for you. If I find the time, I'll accompany you to the hospital. 35. Shinji cries easily, you know. 36. Hey, wait up. You're just going to leave this, huh? 37. I won't leave it be, but I'm busy right now. If it's not cancer, then it can wait for a bit. 38. Dysplasia isn't exactly cancer, but it's kind of like the groundwork for it. 39. The groundwork for cancer? 40. It's a little tricky. Let me break down how cervical cancer develops. 41. About 95% of cervical cancer cases are caused by infection with a certain virus. 42. It's called human papillomavirus, also known as HPV. 43. HPV is transmitted primarily through sexual activities. 44. Wait a minute. I've been faithful to her, and I haven't been sexually active with anyone else but her. 45. No way, is Maki having an affair? 46. Yeah. That’s the worst common misconception. 47. HPV is a highly prevalent virus that can affect anyone, including men and women. If an individual has engaged in sexual intercourse at least once in the past, they may be at risk of infection. 48. 80-90% of both men and women are infected with HPV. 49. So, it's a terrible misconception to suspect sexual promiscuity or infidelity just because you have dysplasia or cervical cancer. 50. By the way, HPV can be transmitted even when using a condom. 51. So, does this mean that every woman who has ever had sex would eventually get cervical cancer? 52. Yes. You would think so, wouldn’t you? 53. Condom use is also important to avoid unwanted pregnancy and other infections. Note: The numbers correspond to the translation of the similarly numbered panels.

**Figure 8:**
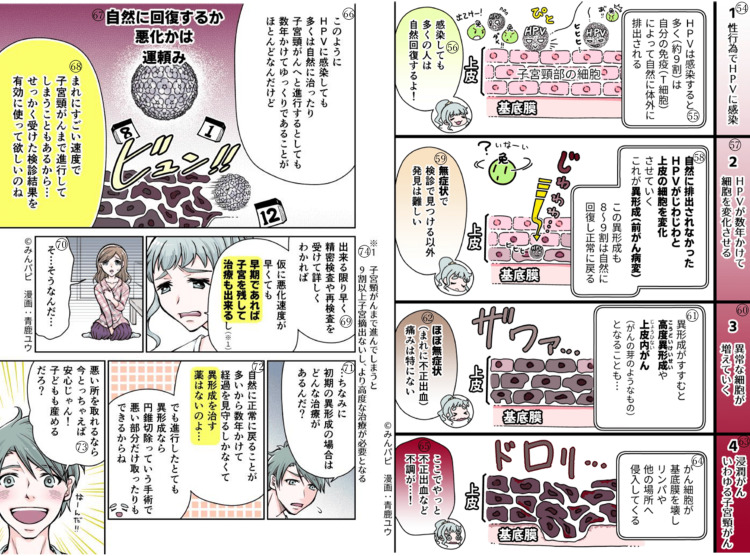
Cervical cancer screening, HPV, and cervical cancer (3/5) 54. HPV is sexually transmitted. 55. Most (90%) HPV infections are cleared by the immune system (T cells). 56. Most infected people recover naturally. 57. HPV changes cells over several years. 58. If not cleared, HPV gradually alters epithelial cells, leading to dysplasia. Fortunately, 80-90% of these dysplasias resolve naturally. 59. It is asymptomatic and difficult to detect without medical exams. 60. Abnormal cells increase in abundance. 61. Dysplasia can progress to severe dysplasia or intraepithelial carcinoma. 62. It is nearly asymptomatic; abnormal genital bleeding is rare. You wouldn’t feel specific pain. 63. Invasive cancer. So-called cervical cancer. 64. Cancer cells invade the basement membrane, lymphatic system, and beyond. 65. At this stage, symptoms like abnormal genital bleeding become noticeable. 66. Most HPV infections resolve naturally or progress slowly over years to cervical cancer. 67. Whether it resolves or worsens depends on luck. 68. Rarely, it progresses quickly, so I want you to make the most of the results of the screening you have received. 69. By undergoing a thorough examination as soon as possible, even if the deterioration rate is rapid, uterus-sparing treatment is possible if the disease is detected early. 70. I see. 71. By the way, what is the treatment for early dysplasia? 72. There is no medication for dysplasia. It often reverts to normal, requiring careful monitoring over years. If advanced but still dysplasia, conization can remove only the affected portion. 73. If you can cut out only the bad parts, why not cut them out now, that would be a relief. Then you can have children, right? 74. If it progresses to cervical cancer, more than 90% of patients will require a hysterectomy or more advanced treatment. Note: The numbers correspond to the translation of the similarly numbered panels.

**Figure 9:**
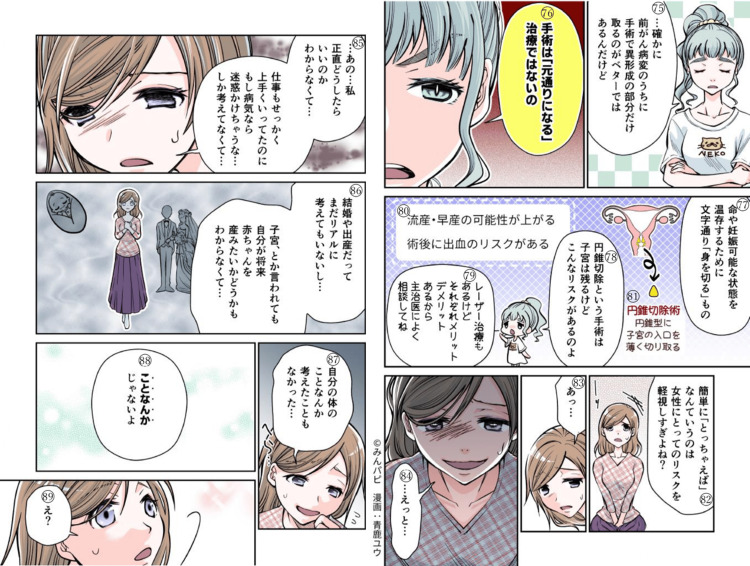
Cervical cancer screening, HPV, and cervical cancer (4/5) 75. Indeed. Undergoing surgical removal of the precancerous area seems like a prudent course of action. 76. But your body would not exactly be the same after surgery. 77. Surgery literally cuts your body to preserve life and fertility. 78. The conization surgery can preserve the uterus but carries the following risks. 79. There is also laser treatment, but each option has its own set of advantages and disadvantages, so consult carefully with your doctor. 80. Increased risk of miscarriage and premature birth. 81. Conization. Excise the cervix in a conical shape. 82. Saying “Why don’t you remove it” so easily is to underestimate the risks to women. 83. Oh. 84. Well. 85. Um, I honestly don't know what to do. My work was going so well, but if I were sick, I would be a nuisance to everyone. 86. I haven't even seriously thought about getting married or having a baby yet. I don't even know if I want to have a baby in the future. 87. I have never thought about my body. 88. You need to take better care of your body. 89. What? Note: The numbers correspond to the translation of the similarly numbered panels.

**Figure 10:**
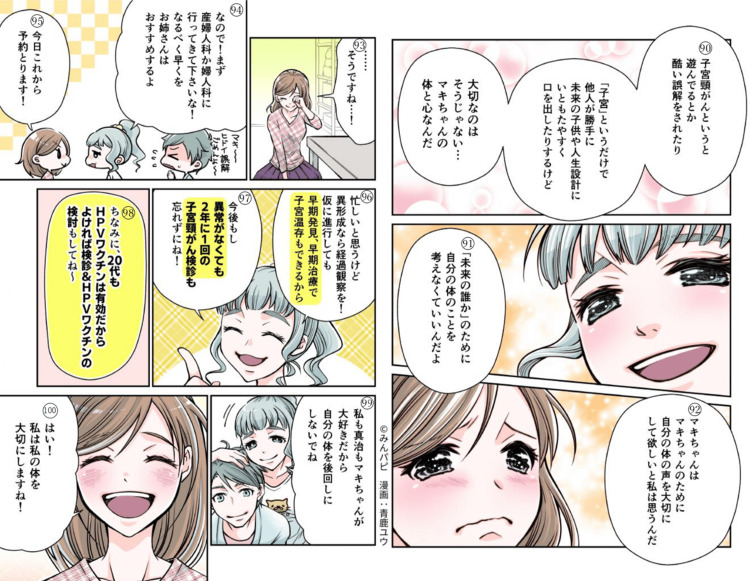
Cervical cancer screening, HPV, and cervical cancer (5/5) 90. When it comes to cervical cancer, people often have a terrible misconception that it is linked to sexual promiscuity. And, it is a matter of the uterus, so it is easy for others to interfere with birth and life planning, but what is important is your body and your mind. 91. You don't have to think about your body for someone else in the future. 92. I believe it's important for you to listen to your own body and take care of its needs. 93. Right. 94. So please see an obstetrician/gynecologist first. I recommend that you do so as soon as possible. 95. I will make an appointment today. 96. I know you are busy, but if it is dysplasia, please have it followed up. 97. Even if there are no abnormalities in the future, don't forget to have a cervical cancer screening every 2 years. 98. By the way, the HPV vaccine is effective for people in their 20s, so consider getting screened regularly and getting the HPV vaccine if you want. 99. Shinji and I both love you, so please don't neglect your own well-being. 100. Sure! I will take good care of my body! Note: The numbers correspond to the translation of the similarly numbered panels.

## Appendices

Appendix 1 

**Table 8:** Questionnaire.

Questions asked only before the distribution of the educational materials (for males and females)		
	1. Please provide your birth year and month, as well as your sex.	
	2. What is your current living situation? Options: Living alone, living with parents, living with others (not parents).	
	3. What is your current marital status? Options: Married, Single (in a relationship), Single (not in a relationship).	
Questions asked both before and after the distribution of the educational materials (for males and females)		
	1. Please select the items you think are related to the development of cancer. (Multiple answers allowed)	
		a. Hepatitis B virus (HBV)
		b. Hepatitis C virus (HCV)
		c. Human immunodeficiency virus (HIV)
		d. Human papillomavirus (HPV)
		e. Helicobacter pylori
		f. Not sure
Questions about human papillomavirus (HPV)		
	2. Which sexes can be affected by human papillomavirus (HPV) or develop related diseases?	
		a. Both men and women
		b. Only men
		c. Only women
		d. Not sure
	3. What is the primary mode of transmission for human papillomavirus (HPV)?	
		a. Inhalation (airborne)
		b. Sexual contact
		c. Consumption of food and drink
		d. Not sure
	4. Who is eligible to receive a publicly funded (free) HPV vaccine for human papillomavirus (HPV) infection?	
		a. Boys and girls aged between 12 and 16 years
		b. Boys aged between 12 and 16 years
		c. Girls aged between 12 and 16 years
		d. Anyone
		e. Not sure
	5. Are you familiar with catch-up vaccination for the HPV vaccine?	
		a. Yes, I’m very familiar with it.
		b. Yes, I know about it.
		c. I’ve heard of it, but I’m not very knowledgeable.
		d. No, I have no idea.
		e. Not sure
Questions asked both before and after the distribution of the educational materials (for males)		
	1. Have you received the HPV vaccine?	
		a. Yes, I have completed the series of vaccinations.
		b. Yes, but I have not completed the series.
		c. No, but I would like to be vaccinated in the future.
		d. No, and I do not wish to be vaccinated in the future.
		e. Not sure
Questions about oropharyngeal cancer, anal cancer, and condyloma acuminatum		
	2. Do you think you could be at risk of developing them?	
		a. I strongly agree.
		b. I somewhat agree.
		c. I’m uncertain.
		d. I somewhat disagree.
		e. I strongly disagree.
	3. Do you think that some of these are life-threatening diseases?	
		a. I strongly agree.
		b. I somewhat agree.
		c. I’m uncertain.
		d. I somewhat disagree.
		e. I strongly disagree.
	4. Do you think that they are preventable with the HPV vaccine?	
		a. I strongly agree.
		b. I somewhat agree.
		c. I’m uncertain.
		d. I somewhat disagree.
		e. I strongly disagree.
	5. Would you receive the HPV vaccine if it were free or cheaper?	
		a. I strongly agree.
		b. I somewhat agree.
		c. I’m uncertain.
		d. I somewhat disagree.
		e. I strongly disagree.
Questions asked both before and after the distribution of the educational materials (for females)		
	1. Have you received the HPV vaccine?	
		a. Yes, I have completed the series of vaccinations.
		b. Yes, but I have not completed the series.
		c. No, but I would like to be vaccinated in the future.
		d. No, and I do not wish to be vaccinated in the future.
		e. Not sure
	2. Have you ever undergone screening for cervical cancer?	
		a. Yes, I undergo regular screening.
		b. Yes, I have undergone screening before.
		c. No, but I would like to undergo screening in the future.
		d. No, and I do not wish to undergo screening in the future.
		e. Not sure.
Questions about cervical cancer		
	3. Do you think you could be at risk of developing it?	
		a. I strongly agree.
		b. I somewhat agree.
		c. I’m uncertain.
		d. I somewhat disagree.
		e. I strongly disagree.
	4. Do you think that it is a life-threatening disease?	
		a. I strongly agree.
		b. I somewhat agree.
		c. I’m uncertain.
		d. I somewhat disagree.
		e. I strongly disagree.
	5. Do you think that developing cervical cancer means you cannot conceive?	
		a. I strongly agree.
		b. I somewhat agree.
		c. I’m uncertain.
		d. I somewhat disagree.
		e. I strongly disagree.
	6. Do you think that the HPV vaccine is effective in preventing cervical cancer?	
		a. I strongly agree.
		b. I somewhat agree.
		c. I’m uncertain.
		d. I somewhat disagree.
		e. I strongly disagree.
	7. Do you think that cervical cancer screening can prevent or detect cervical cancer in its early stages?	
		a. I strongly agree.
		b. I somewhat agree.
		c. I’m uncertain.
		d. I somewhat disagree.
		e. I strongly disagree.
	8. Do you think that you have concerns about getting the HPV vaccine?	
		a. I strongly agree.
		b. I somewhat agree.
		c. I’m uncertain.
		d. I somewhat disagree.
		e. I strongly disagree.
	9. Do you think that you feel hesitant or resistant to visiting an OB/GYN for cervical cancer screening?	
		a. I strongly agree.
		b. I somewhat agree.
		c. I’m uncertain.
		d. I somewhat disagree.
		e. I strongly disagree.
	10. Do you think that you are unwilling to undergo cervical cancer screening because you do not know what kind of tests will be performed?	
		a. I strongly agree.
		b. I somewhat agree.
		c. I’m uncertain.
		d. I somewhat disagree.
		e. I strongly disagree.
	11. Do you think that undergoing regular cervical cancer screening is a hassle?	
		a. I strongly agree.
		b. I somewhat agree.
		c. I’m uncertain.
		d. I somewhat disagree.
		e. I strongly disagree.
Questions asked only after the education (for male and female)		
	12. Are the educational materials distributed easy for you to understand?	
		a. Very easy to understand.
		b. Easy to understand.
		c. Neither easy nor difficult.
		d. A little difficult to understand.
		e. Very difficult to understand.
		f. I haven’t read them at all.

Appendix 2 

Appendix 3 

Appendix 4 

Appendix 5 

Appendix 6 

Appendix 7 

Appendix 8 

Appendix 9 

Appendix 10 

## References

[1] Sung H, Ferlay J, Siegel RL, Laversanne M, Soerjomataram I, Jemal A, Bray F (2021). Global Cancer Statistics 2020: GLOBOCAN estimates of incidence and mortality worldwide for 36 cancers in 185 countries. CA Cancer J Clin.

[2] de Sanjose S, Quint WG, Alemany L (2010). Human papillomavirus genotype attribution in invasive cervical cancer: a retrospective cross-sectional worldwide study. Lancet Oncol.

[3] Sawada M, Ueda Y, Yagi A (2018). HPV vaccination in Japan: results of a 3-year follow-up survey of obstetricians and gynecologists regarding their opinions toward the vaccine. Int J Clin Oncol.

[4] Hanley SJ, Yoshioka E, Ito Y (2015). HPV vaccination crisis in Japan. Lancet.

[5] Nakagawa S, Ueda Y, Yagi A, Ikeda S, Hiramatsu K, Kimura T (2020). Corrected human papillomavirus vaccination rates for each birth fiscal year in Japan. Cancer Sci.

[6] Morimoto A, Ueda Y, Egawa-Takata T (2015). Effect on HPV vaccination in Japan resulting from news report of adverse events and suspension of governmental recommendation for HPV vaccination. Int J Clin Oncol.

[7] Masuda T, Ueda Y, Kimura T (2020). Consideration on the study for safety of human papillomavirus(es) vaccine in Japan. J Obstet Gynaecol Res.

[8] Fukushima W, Hara M, Kitamura Y (2022). A nationwide epidemiological survey of adolescent patients with diverse symptoms similar to those following human papillomavirus vaccination: background prevalence and incidence for considering vaccine safety in Japan. J Epidemiol.

[9] Sabatino SA, Thompson TD, White MC, Villarroel MA, Shapiro JA, Croswell JM, Richardson LC (2023). Up-to-date breast, cervical, and colorectal cancer screening test use in the United States, 2021. Prev Chronic Dis.

[10] Mitoma T, Maki J, Ooba H, Ogawa C, Masuyama H, Tabuchi T (2024). Association of regular cervical cancer screening with socioeconomic, COVID-19 infection and vaccine status among Japanese population: cohort observational study. Int J Gen Med.

[11] Harris PA, Taylor R, Minor BL (2019). The REDCap consortium: building an international community of software platform partners. J Biomed Inform.

[12] Harris PA, Taylor R, Thielke R, Payne J, Gonzalez N, Conde JG (2009). Research electronic data capture (REDCap)--a metadata-driven methodology and workflow process for providing translational research informatics support. J Biomed Inform.

[13] Janz NK, Becker MH (1984). The Health Belief Model: a decade later. Health Educ Q.

[14] Miyoshi A, Ueda Y, Yagi A (2021). Educational intervention for women in Japan coming of age for cervical cancer screening who grew up during the suspended HPV-vaccination-program. Hum Vaccin Immunother.

[15] Leung SO, Villa A, Duffey-Lind E, Welch K, Jabaley T, Hammer M, Feldman S (2023). An interactive educational tool to improve human papillomavirus vaccine knowledge and recommendation among nurses. J Cancer Educ.

[16] Suzuki Y, Sukegawa A, Ueda Y (2022). The effect of a web-based cervical cancer survivor's story on parents' behavior and willingness to consider human papillomavirus vaccination for daughters: randomized controlled trial. JMIR Public Health Surveill.

[17] Ampofo AG, Boyes AW, Khumalo PG, Mackenzie L (2022). Improving knowledge, attitudes, and uptake of cervical cancer prevention among female students: a systematic review and meta-analysis of school-based health education. Gynecol Oncol.

[18] Fisher H, Chantler T, Finn A (2022). Development of an educational package for the universal human papillomavirus (HPV) vaccination programme: a co-production study with young people and key informants. Res Involv Engagem.

[19] Thiel de Bocanegra H, Dehlendorf C, Kuppermann M, Vangala SS, Moscicki AB (2022). Impact of an educational tool on young women's knowledge of cervical cancer screening recommendations. Cancer Causes Control.

[20] Somera LP, Diaz T, Mummert A, Badowski G, Choi J, Palaganas H, Ayson K (2023). Cervical cancer and HPV knowledge and awareness: an educational intervention among college students in Guam. Asian Pac J Cancer Prev.

[21] Shin HY, Song SY, Jun JK, Kim KY, Kang P (2021). Barriers and strategies for cervical cancer screening: What do female university students know and want?. PLoS One.

[22] Dönmez S, Öztürk R, Kısa S, Karaoz Weller B, Zeyneloğlu S (2019). Knowledge and perception of female nursing students about human papillomavirus (HPV), cervical cancer, and attitudes toward HPV vaccination. J Am Coll Health.

[23] Hayes KN, Pan I, Kunkel A, McGivney MS, Thorpe CT (2019). Evaluation of targeted human papillomavirus vaccination education among undergraduate college students. J Am Coll Health.

